# 1-(6-Chloro-1,3-benzothia­zol-2-yl)hydrazine

**DOI:** 10.1107/S1600536812005442

**Published:** 2012-02-17

**Authors:** Hoong-Kun Fun, Chin Wei Ooi, B. K. Sarojini, B. J. Mohan, B. Narayana

**Affiliations:** a X-ray Crystallography Unit, School of Physics, Universiti Sains Malaysia, 11800 USM, Penang, Malaysia; b Department of Chemistry, P. A. College of Engineering, Mangalore 574 153, India; c Department of Chemistry, Mangalore University, Mangalagangotri 574 199, Mangalore India

## Abstract

The asymmetric unit of the title compound, C_7_H_6_ClN_3_S, consists of two crystallographically independent mol­ecules (*A* and *B*). The dihedral angle between the benzothia­zole ring system and the hydrazine group is 8.71 (6)° in mol­ecule *A* and 7.16 (6)° in mol­ecule *B*. The N—N—C—N and N—N—C—S torsion angles involving the hydrazine group are 170.89 (9) and −9.96 (13)°, respectively, in mol­ecule *A* and 172.50 (9) and −7.43 (13)°, respectively, in mol­ecule *B*. In the crystal, neighbouring mol­ecules are connected *via* pairs of N—H⋯N hydrogen bonds, generating *R*_2_^2^(8) ring motifs, and are connected further by N—H⋯N hydrogen bonds into sheets lying parallel to the *ab* plane. The crystal studied was an inversion twin, the refined ratio of the twin components being 0.50 (3):0.50 (3).

## Related literature

For the biological activity of benzothia­zole derivatives, see: Bowyer *et al.* (2007 ▶); Gurupadayya *et al.* (2008 ▶); Kini *et al.* (2007 ▶); Mittal *et al.* (2007 ▶); Munirajasekhar *et al.* (2011 ▶); Rana *et al.* (2008 ▶); Pozas *et al.* (2005 ▶); Yaseen *et al.* (2006 ▶). For hydrogen-bond motifs, see: Bernstein *et al.* (1995 ▶). For related structures, see: Fun *et al.* (2011*a* ▶,*b* ▶,*c* ▶,*d* ▶). For bond-length data, see: Allen *et al.* (1987 ▶). For the stability of the temperature controller used for data collection, see: Cosier & Glazer (1986 ▶).
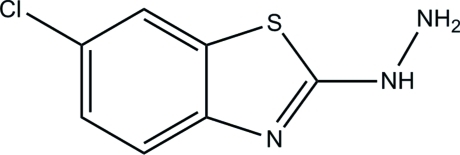


## Experimental

### 

#### Crystal data

C_7_H_6_ClN_3_S
*M* *_r_* = 199.66
Orthorhombic, 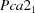

*a* = 13.0225 (13) Å
*b* = 5.7767 (6) Å
*c* = 21.708 (2) Å
*V* = 1633.0 (3) Å^3^
*Z* = 8
Mo *K*α radiation
μ = 0.66 mm^−1^
*T* = 100 K
0.46 × 0.33 × 0.22 mm

#### Data collection

Bruker APEX DUO CCD area-detector diffractometer
Absorption correction: multi-scan (*SADABS*; Bruker, 2009 ▶) *T*_min_ = 0.752, *T*_max_ = 0.867
12527 measured reflections
5771 independent reflections
5686 reflections with *I* > 2σ(*I*)
*R*_int_ = 0.015

#### Refinement

*R*[*F* ^2^ > 2σ(*F* ^2^)] = 0.019
*wR*(*F* ^2^) = 0.052
*S* = 1.04
5771 reflections
242 parameters
1 restraint
H atoms treated by a mixture of independent and constrained refinement
Δρ_max_ = 0.39 e Å^−3^
Δρ_min_ = −0.21 e Å^−3^
Absolute structure: Flack (1983 ▶), with 2734 Friedel pairs
Flack parameter: 0.50 (3)

### 

Data collection: *APEX2* (Bruker, 2009 ▶); cell refinement: *SAINT* (Bruker, 2009 ▶); data reduction: *SAINT*; program(s) used to solve structure: *SHELXTL* (Sheldrick, 2008 ▶); program(s) used to refine structure: *SHELXTL*; molecular graphics: *SHELXTL*; software used to prepare material for publication: *SHELXTL* and *PLATON* (Spek, 2009 ▶).

## Supplementary Material

Crystal structure: contains datablock(s) global, I. DOI: 10.1107/S1600536812005442/lh5415sup1.cif

Structure factors: contains datablock(s) I. DOI: 10.1107/S1600536812005442/lh5415Isup2.hkl

Supplementary material file. DOI: 10.1107/S1600536812005442/lh5415Isup3.cml

Additional supplementary materials:  crystallographic information; 3D view; checkCIF report

## Figures and Tables

**Table 1:** Hydrogen-bond geometry (Å, °)

*D*—H⋯*A*	*D*—H	H⋯*A*	*D*⋯*A*	*D*—H⋯*A*
N2*A*—H1N2⋯N1*B*^i^	0.89 (2)	2.03 (2)	2.9084 (12)	170.5 (18)
N2*B*—H2N2⋯N1*A*^ii^	0.897 (17)	2.059 (18)	2.9539 (13)	175.3 (16)
N3*A*—H1N3⋯N3*B*^iii^	0.831 (18)	2.53 (2)	3.1776 (13)	135.6 (16)
N3*B*—H3N3⋯N3*A*	0.863 (16)	2.435 (17)	3.1383 (13)	139.1 (14)

Symmetry codes: (i) 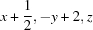; (ii) 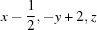; (iii) 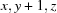.

**Fig. 1.**
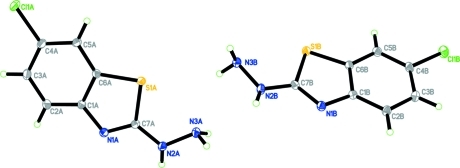
The molecular structure of the title compound, showing 50% probability displacement ellipsoids and the atom-numbering scheme.

**Fig. 2.**
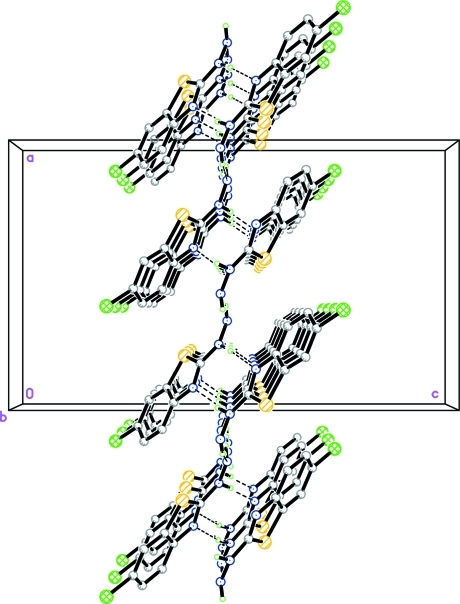
The crystal packing of the title compound, viewed along the b axis. H atoms not involved in the intermolecular interactions (dashed lines) have been omitted for clarity.

## supplementary crystallographic information

### Comment

Benzothiazoles are very important bicyclic ring compounds which are of great interest because of their biological activities. The substituted benzothiazole derivatives have emerged as significant components in various diversified therapeutic applications. The literature review reveals that benzothiazoles and their derivatives show considerable activity, including potent inhibition of human immunodeficiency virus type 1 (HIV-1) replication by HIV-1 protease inhibition (Yaseen *et al.*, 2006), antitumor (Kini *et al.*, 2007), anthelmintic (Munirajasekhar *et al.*, 2011) analgesic and anti-inflammatory (Gurupadayya *et al.*, 2008), antimalarial (Bowyer *et al.*, 2007), antifungal (Mittal *et al.*, 2007), anticandidous activities (Rocío Pozas *et al.*, 2005) and various CNS activities (Rana *et al.*, 2008). The related structures have been reported by Fun *et al.* (2011*a*,*b*,*c*,*d*). The present work describes the synthesis and crystal structure of the title compound, 1-(6-chloro-1,3-benzothiazol-2-yl)hydrazine, which was prepared from the reaction of 2-amino-6-chlorobenzothiazole treated with hydrazine.

The asymmetric unit of the title compound consists of two crystallographically independent molecules (A and B) as shown in Fig. 1. The dihedral angle between the benzothiazole (S1/N1/C1–C7) ring system and the hydrazine (N2A/N3A) group is 8.71 (6)° in molecule A whereas it is equal to 7.16 (6)° in molecule B. The hydrazine group is twisted slightly with N3—N2—C7—N1 and N3—N2—C7—S1 torsion angles of 170.89 (9)°: -9.96 (13)° in molecule A and 172.50 (9)°: -7.43 (13)° in molecule B. The bond lengths (Allen *et al.*, 1987) and angles are within normal ranges and are comparable to the related structure (Fun *et al.*, 2011*a*,*b*,*c*,*d*).

In the crystal structure (Fig. 2), the neighbouring molecules are connected *via* pairs of intermolecular N2A—H1N2···N1B^i^ and N2B—H2N2···N1A^ii^ (Table 1) hydrogen bonds, generating *R*_2_^2^ (8) ring motifs (Bernstein *et al.*, 1995). Furthermore, the molecules are linked into sheets lying parallel to the *ab* plane *via* intermolecular N3B—H3N3···N3A and N3A—H1N3···N3B^iii^ hydrogen bonds.

### Experimental

2-Amino-6-chlorobenzothiazole (5.52 g, 0.03 mol) and hydrazine hydrate (85%) (0.12 mol) in 50 ml of ethylene glycol were refluxed by stirring for 4 h at 333 K. A white solid was precipitated at the end of the reflux period. The mixture was cooled and the product was filtered and then washed with water several times. Then the product was air-dried and recrystallized by using ethanol. The single crystals were grown by slow evaporation from solvent ethanol and dichloromethane (1:1 *v*/*v*) (m.p. 470–472 K).

### Refinement

H1N2, H2N2, H1N3, H2N3, H3N3 and H4N3 were located in a difference Fourier map and were refined freely [N—H = 0.831 (18)–0.968 (19) Å]. The remaining H atoms were positioned geometrically and refined using a riding model with *U*_iso_(H) = 1.2*U*_eq_(C) [C—H = 0.95 Å]. The crystal studied was an inversion twin, the refined ratio of the twin components being 0.50 (3):0.50 (3). In the final refinement, the outliers (5 3 2), (6 0 12), (4 0 4) and (14 0 4) were omitted.

### Crystal data

**Table:**

C_7_H_6_ClN_3_S	*F*(000) = 816
*M**_r_* = 199.66	*D*_x_ = 1.624 Mg m^−^^3^
Orthorhombic, *P**c**a*2_1_	Mo *K*α radiation, λ = 0.71073 Å
Hall symbol: P 2c -2ac	Cell parameters from 9942 reflections
*a* = 13.0225 (13) Å	θ = 3.1–32.6°
*b* = 5.7767 (6) Å	µ = 0.66 mm^−^^1^
*c* = 21.708 (2) Å	*T* = 100 K
*V* = 1633.0 (3) Å^3^	Block, colourless
*Z* = 8	0.46 × 0.33 × 0.22 mm

### Data collection

**Table:**

Bruker APEX DUO CCD area-detector diffractometer	5771 independent reflections
Radiation source: fine-focus sealed tube	5686 reflections with *I* > 2σ(*I*)
Graphite monochromator	*R*_int_ = 0.015
φ and ω scans	θ_max_ = 32.6°, θ_min_ = 3.1°
Absorption correction: multi-scan (*SADABS*; Bruker, 2009)	*h* = −18→19
*T*_min_ = 0.752, *T*_max_ = 0.867	*k* = −8→8
12527 measured reflections	*l* = −32→32

### Refinement

**Table:**

Refinement on *F*^2^	Secondary atom site location: difference Fourier map
Least-squares matrix: full	Hydrogen site location: inferred from neighbouring sites
*R*[*F*^2^ > 2σ(*F*^2^)] = 0.019	H atoms treated by a mixture of independent and constrained refinement
*wR*(*F*^2^) = 0.052	*w* = 1/[σ^2^(*F*_o_^2^) + (0.030*P*)^2^ + 0.2349*P*] where *P* = (*F*_o_^2^ + 2*F*_c_^2^)/3
*S* = 1.04	(Δ/σ)_max_ = 0.001
5771 reflections	Δρ_max_ = 0.39 e Å^−^^3^
242 parameters	Δρ_min_ = −0.21 e Å^−^^3^
1 restraint	Absolute structure: Flack (1983), with 2734 Friedel pairs
Primary atom site location: structure-invariant direct methods	Flack parameter: 0.50 (3)

### Special details

**Table:**

Experimental. The crystal was placed in the cold stream of an Oxford Cryosystems Cobra open-flow nitrogen cryostat (Cosier & Glazer, 1986) operating at 100.0 (1) K
Geometry. All e.s.d.'s (except the e.s.d. in the dihedral angle between two l.s. planes) are estimated using the full covariance matrix. The cell e.s.d.'s are taken into account individually in the estimation of e.s.d.'s in distances, angles and torsion angles; correlations between e.s.d.'s in cell parameters are only used when they are defined by crystal symmetry. An approximate (isotropic) treatment of cell e.s.d.'s is used for estimating e.s.d.'s involving l.s. planes.
Refinement. Refinement of *F*^2^ against ALL reflections. The weighted *R*-factor *wR* and goodness of fit *S* are based on *F*^2^, conventional *R*-factors *R* are based on *F*, with *F* set to zero for negative *F*^2^. The threshold expression of *F*^2^ > σ(*F*^2^) is used only for calculating *R*-factors(gt) *etc*. and is not relevant to the choice of reflections for refinement. *R*-factors based on *F*^2^ are statistically about twice as large as those based on *F*, and *R*-factors based on ALL data will be even larger.

### Fractional atomic coordinates and isotropic or equivalent isotropic displacement parameters (Å^2^)

**Table:**

	*x*	*y*	*z*	*U*_iso_*/*U*_eq_	
Cl1A	0.87218 (2)	0.41653 (5)	0.717085 (12)	0.02134 (5)	
S1A	0.552566 (17)	0.69138 (4)	0.565134 (11)	0.01203 (5)	
N1A	0.66692 (7)	1.05970 (15)	0.54585 (4)	0.01308 (15)	
N2A	0.51172 (7)	1.05780 (16)	0.49160 (4)	0.01507 (16)	
N3A	0.42819 (7)	0.91902 (16)	0.47219 (4)	0.01386 (15)	
C1A	0.71962 (7)	0.91958 (17)	0.58718 (4)	0.01122 (15)	
C2A	0.81645 (8)	0.96993 (18)	0.61154 (5)	0.01342 (17)	
H2AA	0.8505	1.1099	0.6009	0.016*	
C3A	0.86228 (8)	0.81302 (19)	0.65137 (5)	0.01533 (18)	
H3AA	0.9280	0.8454	0.6683	0.018*	
C4A	0.81171 (8)	0.60698 (18)	0.66667 (5)	0.01434 (17)	
C5A	0.71540 (8)	0.55065 (17)	0.64323 (5)	0.01318 (16)	
H5AA	0.6819	0.4103	0.6540	0.016*	
C6A	0.67048 (7)	0.71010 (16)	0.60320 (5)	0.01111 (15)	
C7A	0.57964 (8)	0.96117 (17)	0.53072 (5)	0.01172 (16)	
Cl1B	−0.11478 (2)	−0.03607 (5)	0.236826 (13)	0.02216 (6)	
S1B	0.204579 (17)	0.20760 (4)	0.392197 (11)	0.01240 (5)	
N1B	0.09520 (7)	0.58201 (14)	0.41251 (4)	0.01264 (14)	
N2B	0.24932 (7)	0.56551 (15)	0.46758 (4)	0.01454 (15)	
N3B	0.33266 (7)	0.42272 (15)	0.48545 (4)	0.01366 (15)	
C1B	0.04080 (7)	0.44800 (16)	0.37042 (4)	0.01125 (15)	
C2B	−0.05487 (8)	0.50641 (18)	0.34582 (5)	0.01351 (17)	
H2BA	−0.0869	0.6485	0.3566	0.016*	
C3B	−0.10279 (8)	0.3538 (2)	0.30526 (5)	0.01507 (17)	
H3BA	−0.1683	0.3902	0.2886	0.018*	
C4B	−0.05411 (8)	0.14744 (19)	0.28917 (5)	0.01476 (17)	
C5B	0.04124 (8)	0.08359 (18)	0.31282 (5)	0.01425 (16)	
H5BA	0.0731	−0.0582	0.3016	0.017*	
C6B	0.08771 (7)	0.23707 (17)	0.35371 (4)	0.01162 (15)	
C7B	0.18114 (8)	0.47712 (17)	0.42752 (5)	0.01170 (16)	
H3N3	0.3862 (12)	0.510 (3)	0.4823 (8)	0.020 (4)*	
H1N2	0.5300 (15)	1.169 (4)	0.4657 (9)	0.032 (5)*	
H2N2	0.2275 (13)	0.679 (3)	0.4927 (8)	0.021 (4)*	
H1N3	0.3749 (13)	0.997 (4)	0.4765 (9)	0.026 (5)*	
H2N3	0.4325 (13)	0.893 (3)	0.4319 (8)	0.019 (4)*	
H4N3	0.3195 (15)	0.373 (3)	0.5273 (8)	0.032 (5)*	

### Atomic displacement parameters (Å^2^)

**Table:**

	*U*^11^	*U*^22^	*U*^33^	*U*^12^	*U*^13^	*U*^23^
Cl1A	0.02667 (12)	0.01723 (10)	0.02012 (11)	0.00445 (9)	−0.01095 (10)	0.00160 (9)
S1A	0.01053 (9)	0.01123 (9)	0.01432 (9)	−0.00103 (7)	−0.00095 (8)	0.00210 (8)
N1A	0.0121 (4)	0.0126 (3)	0.0145 (4)	−0.0016 (3)	−0.0014 (3)	0.0022 (3)
N2A	0.0122 (4)	0.0141 (4)	0.0189 (4)	−0.0023 (3)	−0.0038 (3)	0.0051 (3)
N3A	0.0108 (3)	0.0150 (4)	0.0158 (4)	−0.0001 (3)	−0.0018 (3)	−0.0004 (3)
C1A	0.0106 (4)	0.0124 (4)	0.0107 (4)	0.0002 (3)	0.0000 (3)	0.0002 (3)
C2A	0.0116 (4)	0.0150 (4)	0.0137 (4)	−0.0014 (3)	−0.0007 (3)	−0.0004 (3)
C3A	0.0140 (4)	0.0175 (5)	0.0144 (4)	0.0004 (3)	−0.0034 (3)	−0.0016 (3)
C4A	0.0162 (4)	0.0145 (4)	0.0123 (4)	0.0033 (3)	−0.0036 (3)	0.0001 (3)
C5A	0.0156 (4)	0.0115 (4)	0.0124 (4)	0.0017 (3)	−0.0018 (3)	0.0009 (3)
C6A	0.0106 (4)	0.0112 (4)	0.0116 (4)	−0.0002 (3)	−0.0004 (3)	−0.0003 (3)
C7A	0.0113 (4)	0.0117 (4)	0.0122 (4)	0.0007 (3)	0.0001 (3)	0.0013 (3)
Cl1B	0.02443 (12)	0.02047 (12)	0.02156 (12)	−0.00247 (9)	−0.01001 (10)	−0.00638 (9)
S1B	0.01040 (9)	0.01177 (9)	0.01504 (10)	0.00135 (7)	−0.00118 (8)	−0.00246 (8)
N1B	0.0123 (3)	0.0124 (3)	0.0132 (3)	0.0009 (3)	−0.0013 (3)	−0.0035 (3)
N2B	0.0113 (3)	0.0148 (4)	0.0175 (4)	0.0021 (3)	−0.0040 (3)	−0.0052 (3)
N3B	0.0109 (3)	0.0148 (4)	0.0154 (4)	−0.0004 (3)	−0.0020 (3)	0.0011 (3)
C1B	0.0109 (4)	0.0120 (4)	0.0109 (4)	−0.0001 (3)	0.0007 (3)	−0.0012 (3)
C2B	0.0119 (4)	0.0150 (4)	0.0137 (4)	0.0016 (3)	−0.0004 (3)	−0.0017 (3)
C3B	0.0124 (4)	0.0184 (4)	0.0143 (4)	−0.0001 (3)	−0.0024 (3)	−0.0005 (3)
C4B	0.0161 (4)	0.0154 (4)	0.0129 (4)	−0.0033 (3)	−0.0027 (3)	−0.0025 (3)
C5B	0.0152 (4)	0.0134 (4)	0.0141 (4)	−0.0005 (3)	−0.0011 (3)	−0.0029 (3)
C6B	0.0113 (4)	0.0116 (4)	0.0119 (4)	0.0003 (3)	0.0002 (3)	−0.0008 (3)
C7B	0.0114 (4)	0.0112 (4)	0.0126 (4)	−0.0013 (3)	0.0003 (3)	−0.0016 (3)

### Geometric parameters (Å, º)

**Table:**

Cl1A—C4A	1.7402 (10)	Cl1B—C4B	1.7434 (10)
S1A—C6A	1.7471 (10)	S1B—C6B	1.7445 (10)
S1A—C7A	1.7639 (10)	S1B—C7B	1.7621 (10)
N1A—C7A	1.3129 (13)	N1B—C7B	1.3137 (13)
N1A—C1A	1.3896 (13)	N1B—C1B	1.3913 (13)
N2A—C7A	1.3473 (13)	N2B—C7B	1.3437 (13)
N2A—N3A	1.4154 (13)	N2B—N3B	1.4173 (13)
N2A—H1N2	0.89 (2)	N2B—H2N2	0.897 (17)
N3A—H1N3	0.831 (18)	N3B—H3N3	0.862 (17)
N3A—H2N3	0.890 (17)	N3B—H4N3	0.968 (19)
C1A—C2A	1.3979 (14)	C1B—C2B	1.3968 (14)
C1A—C6A	1.4124 (14)	C1B—C6B	1.4106 (13)
C2A—C3A	1.3877 (15)	C2B—C3B	1.3935 (14)
C2A—H2AA	0.9500	C2B—H2BA	0.9500
C3A—C4A	1.4002 (15)	C3B—C4B	1.3946 (15)
C3A—H3AA	0.9500	C3B—H3BA	0.9500
C4A—C5A	1.3921 (15)	C4B—C5B	1.3934 (15)
C5A—C6A	1.3948 (14)	C5B—C6B	1.3929 (14)
C5A—H5AA	0.9500	C5B—H5BA	0.9500
			
C6A—S1A—C7A	88.28 (5)	C6B—S1B—C7B	88.34 (5)
C7A—N1A—C1A	109.67 (9)	C7B—N1B—C1B	109.88 (8)
C7A—N2A—N3A	117.22 (8)	C7B—N2B—N3B	117.51 (8)
C7A—N2A—H1N2	121.6 (13)	C7B—N2B—H2N2	117.4 (11)
N3A—N2A—H1N2	115.4 (13)	N3B—N2B—H2N2	120.0 (11)
N2A—N3A—H1N3	107.6 (13)	N2B—N3B—H3N3	105.0 (12)
N2A—N3A—H2N3	109.9 (11)	N2B—N3B—H4N3	107.2 (12)
H1N3—N3A—H2N3	104.8 (17)	H3N3—N3B—H4N3	113.0 (17)
N1A—C1A—C2A	124.65 (9)	N1B—C1B—C2B	124.81 (9)
N1A—C1A—C6A	115.73 (9)	N1B—C1B—C6B	115.41 (9)
C2A—C1A—C6A	119.59 (9)	C2B—C1B—C6B	119.78 (9)
C3A—C2A—C1A	119.20 (9)	C3B—C2B—C1B	119.23 (9)
C3A—C2A—H2AA	120.4	C3B—C2B—H2BA	120.4
C1A—C2A—H2AA	120.4	C1B—C2B—H2BA	120.4
C2A—C3A—C4A	120.04 (9)	C2B—C3B—C4B	119.69 (9)
C2A—C3A—H3AA	120.0	C2B—C3B—H3BA	120.2
C4A—C3A—H3AA	120.0	C4B—C3B—H3BA	120.2
C5A—C4A—C3A	122.39 (9)	C5B—C4B—C3B	122.63 (9)
C5A—C4A—Cl1A	119.33 (8)	C5B—C4B—Cl1B	118.88 (8)
C3A—C4A—Cl1A	118.28 (8)	C3B—C4B—Cl1B	118.49 (8)
C4A—C5A—C6A	116.82 (9)	C6B—C5B—C4B	116.96 (9)
C4A—C5A—H5AA	121.6	C6B—C5B—H5BA	121.5
C6A—C5A—H5AA	121.6	C4B—C5B—H5BA	121.5
C5A—C6A—C1A	121.95 (9)	C5B—C6B—C1B	121.71 (9)
C5A—C6A—S1A	128.49 (8)	C5B—C6B—S1B	128.47 (8)
C1A—C6A—S1A	109.56 (7)	C1B—C6B—S1B	109.81 (7)
N1A—C7A—N2A	123.12 (9)	N1B—C7B—N2B	123.24 (9)
N1A—C7A—S1A	116.75 (8)	N1B—C7B—S1B	116.56 (8)
N2A—C7A—S1A	120.12 (7)	N2B—C7B—S1B	120.20 (7)
			
C7A—N1A—C1A—C2A	178.40 (10)	C7B—N1B—C1B—C2B	178.69 (10)
C7A—N1A—C1A—C6A	0.22 (12)	C7B—N1B—C1B—C6B	0.05 (12)
N1A—C1A—C2A—C3A	−178.45 (9)	N1B—C1B—C2B—C3B	−178.22 (9)
C6A—C1A—C2A—C3A	−0.33 (15)	C6B—C1B—C2B—C3B	0.37 (15)
C1A—C2A—C3A—C4A	0.10 (16)	C1B—C2B—C3B—C4B	−0.92 (15)
C2A—C3A—C4A—C5A	0.06 (16)	C2B—C3B—C4B—C5B	0.97 (16)
C2A—C3A—C4A—Cl1A	−179.72 (8)	C2B—C3B—C4B—Cl1B	−178.42 (8)
C3A—C4A—C5A—C6A	0.02 (15)	C3B—C4B—C5B—C6B	−0.41 (16)
Cl1A—C4A—C5A—C6A	179.80 (8)	Cl1B—C4B—C5B—C6B	178.97 (8)
C4A—C5A—C6A—C1A	−0.26 (15)	C4B—C5B—C6B—C1B	−0.17 (15)
C4A—C5A—C6A—S1A	178.95 (8)	C4B—C5B—C6B—S1B	178.78 (8)
N1A—C1A—C6A—C5A	178.70 (9)	N1B—C1B—C6B—C5B	178.90 (9)
C2A—C1A—C6A—C5A	0.42 (15)	C2B—C1B—C6B—C5B	0.18 (15)
N1A—C1A—C6A—S1A	−0.64 (11)	N1B—C1B—C6B—S1B	−0.22 (11)
C2A—C1A—C6A—S1A	−178.92 (8)	C2B—C1B—C6B—S1B	−178.94 (8)
C7A—S1A—C6A—C5A	−178.65 (10)	C7B—S1B—C6B—C5B	−178.81 (10)
C7A—S1A—C6A—C1A	0.64 (8)	C7B—S1B—C6B—C1B	0.24 (8)
C1A—N1A—C7A—N2A	179.49 (9)	C1B—N1B—C7B—N2B	−179.78 (9)
C1A—N1A—C7A—S1A	0.32 (11)	C1B—N1B—C7B—S1B	0.15 (11)
N3A—N2A—C7A—N1A	170.89 (9)	N3B—N2B—C7B—N1B	172.50 (10)
N3A—N2A—C7A—S1A	−9.96 (13)	N3B—N2B—C7B—S1B	−7.43 (13)
C6A—S1A—C7A—N1A	−0.58 (8)	C6B—S1B—C7B—N1B	−0.23 (9)
C6A—S1A—C7A—N2A	−179.78 (9)	C6B—S1B—C7B—N2B	179.70 (9)

### Hydrogen-bond geometry (Å, º)

**Table:**

*D*—H···*A*	*D*—H	H···*A*	*D*···*A*	*D*—H···*A*
N2*A*—H1*N*2···N1*B*^i^	0.89 (2)	2.03 (2)	2.9084 (12)	170.5 (18)
N2*B*—H2*N*2···N1*A*^ii^	0.897 (17)	2.059 (18)	2.9539 (13)	175.3 (16)
N3*A*—H1*N*3···N3*B*^iii^	0.831 (18)	2.53 (2)	3.1776 (13)	135.6 (16)
N3*B*—H3*N*3···N3*A*	0.863 (16)	2.435 (17)	3.1383 (13)	139.1 (14)

Symmetry codes: (i) *x*+1/2, −*y*+2, *z*; (ii) *x*−1/2, −*y*+2, *z*; (iii) *x*, *y*+1, *z*.
